# Plan quality analysis of stereotactic ablative body radiotherapy treatment planning in liver tumor

**DOI:** 10.1002/acm2.13948

**Published:** 2023-03-01

**Authors:** Anirut Watcharawipha, Somvilai Chakrabandhu, Anupong Kongsa, Damrongsak Tippanya, Imjai Chitapanarux

**Affiliations:** ^1^ Division of Radiation Oncology Department of Radiology Faculty of Medicine, Chiang Mai University Chiang Mai Thailand; ^2^ Northern Thai Research Group of Radiation Oncology (NTRG‐RO) Faculty of Medicine, Chiang Mai University Chiang Mai Thailand; ^3^ Chiang Mai Cancer Registry Maharaj Nakorn Chiang Mai Hospital Faculty of Medicine, Chiang Mai University Chiang Mai Thailand

**Keywords:** helical tomotherapy, liver tumor, stereotactic ablative body radiotherapy, volumetric‐modulated arc therapy

## Abstract

**Purpose:**

Stereotactic ablative body radiotherapy (SABR) in the liver, RTOG‐1112 guides the treatment modalities including the dose constraints for this technique but not the plan parameters. This study is not only analyzing the plan quality by utilizing the plan parameters and indexes but also compares treatment modalities from the protocol implementation.

**Method and material:**

Twenty‐five patients treated in the period from February 2020 to September 2022 were recruited in this analysis. Two planners randomly selected the patients and modalities. The modalities employed were Volumetric‐Modulated Arc Therapy (VMAT) and Helical Tomotherapy (HT). Various parameters and indexes were used to access not only the plan quality but also to compare each modality. The parameters and indexes studied were the homogeneity index (HI), conformity index (CI), gradient distance (GD), and the dose received by the organs at risk.

**Result:**

The data reveals that the mean volume of PTV is 60.8 ± 53.9 cc where these targets exhibit no significant difference between each modality. The HI shows a consistent value for both modalities. Between each modality, the CI value shows less deviation, but the HT shows slightly higher performance than VMAT. The value of GD is 1.5 ± 0.3 cm where the HT provides a shorter distance compared to VMAT as well.

**Conclusion:**

The parameters and indexes should be utilized for the plan evaluation although in the guidelines this was not required. Various modalities were employed for treatment. Both can achieve the treatment criteria with slightly low performance of VMAT.

## 1 INTRODUCTION

Radiation therapy for the liver is a challenging treatment process. This technique is the delivery of the high radiation dose to the target in a few fractions. The treatment has to consider not only the surrounding normal organs but also the volume of the liver itself. For the prevention of normal tissue injury, the conventional fractionation is the traditional dose schematic (2 Gy per fraction) for this treatment.^1^ Recently, delivery techniques were developed and utilized to escalate the radiation dose on the tumor. The sophisticated techniques are then widely used are such as three‐dimensional conformal radiotherapy (3D‐CRT),^1^, ^2^ Dynamic conformal arc therapy (DCAT),^3^ Intensity‐modulated radiotherapy (IMRT),^1^, ^4^ Volumetric‐modulated arc therapy (VMAT),^1^, ^5^, ^6^, ^7^ Helical Tomotherapy (HT),^1^, ^8^, ^9^ and CyberKnife (CK).^1^, ^5^, ^7^ These techniques not only increase the radiation dose to the target but also reduce the dose to the normal tissue as well. Particularly, the image‐guided device was equipped with the treatment modality to verify the patient's position during treatment and the technique is termed “Image Guided Radiation Therapy (IGRT)”. Accordingly, the hypofractionation technique then became an attractive treatment for liver tumors termed as Stereotactic Body Radiotherapy (SBRT) or Stereotactic Ablative Body Radiotherapy (SABR).

SABR is the treatment technique that delivers a high radiation dose maintaining a highly accurate tumor position. This technique is utilized not only for lung tumors^10^, ^11^ but also for liver tumors.^1^, ^12^ For the liver tumor, the treatment protocol was guided by the Radiation Therapy Oncology Group No. 1112^1^ (RTOG‐1112). Our center implemented the SABR in the liver tumor and has followed this guideline since February 2020. The RTOG‐1112 is recommended along the treatment modalities of use, the number of fractions, fraction dose for target, limiting dose on organs at risk (OARs), etc. However, this guideline does not mention the plan quality check parameters such as the homogeneity index (HI), conformity index (CI), radiation dose gradient, integral dose (ID), etc.

Since the protocol was implemented, the treatment modalities were utilized by the Linear accelerator (Linac) and Tomotherapy. The treatment plans were performed by the two medical physicists and limited the dose constraints as per the recommendation of the RTOG‐1112. These dose constraints focused only on the radiation dose on the target and OARs but have not considered other plan parameters and quality indexes. This study then observed the plan qualities of the SABR in the liver tumor as well as the dosimetric comparison between each employed treatment modality.

## 2 METHODS AND MATERIALS

### 2.1 Ethical clearance

The study recruited the data from the patient's treatment planning. The information was collected from patients who had been treated with the SABR technique from February 2020 to September 2022. This retrospective study was declared and approved by the Ethic Committee of Chiang Mai University. (Study code: RAD‐2565‐09306)

### 2.2 Simulation and organ delineation

Treatment planning was performed on the patient who was in the inclusion criteria and required the treatment of the SABR technique for liver tumors. The Wing‐board (Standard Board^TM^, CIVCO radiotherapy, USA) was employed as the immobilization. The forced shallow breathing method with an abdominal compressor (Body Pro‐Lok ONE^TM^, CIVCO radiotherapy, USA) was utilized for motion management. The image set of a Computed Tomography (CT) with 3–5 mm of slice thickness was acquired from the Computed Tomography simulator (SOMATOM Definition AS, Siemens Inc., Healthineers, Germany). The sequence of the image acquisition started from an image set of free shallow breathing without contrast, image sets of four‐dimensional CT (4DCT) with contrast, and an image set of free shallow breathing of post‐contrast, respectively. The treatment plan was performed on the image set of free shallow breathing without contrast, whereas the Internal Target Volume (ITV) was delineated by utilizing the Maximum Intensity Projection (MIP) method on the image sets of 4DCT and the image set of free shallow breathing of post‐contrast. The Planning Target Volume (PTV) was 5 mm expanded from the ITV.

### 2.3 Treatment planning

The data were recruited from the patients who had been irradiated with SABR in the period from February 2020 to September 2022. Two treatment modalities were employed for the treatment that consists of the C‐arm‐based Linac (Synergy, Elekta Inc., Crawley, UK) and Ring‐based Linac (Hi‐Art and Radixact, Tomotherapy, Accuray Inc., Wisconsin, USA). The patients were not only treated by randomly selected modalities but also planned by random treatment planners. The data revealed that 14 patients were treated by VMAT, whereas 11 patients were treated by HT. The plans of VMAT were performed by Monaco version 5.11 (Elekta Inc., Missouri, USA), whereas the plans of HT were established by Hi‐Art version 5.1.4 (Accuray Inc., Sunnyvale, California, USA) and Precision version 2.0.1.1 (Accuray Inc., Sunnyvale, California, USA). Monaco performed the dose calculation of the VMAT treatment plans with the dose calculation grid of 3.0 mm by utilizing the Monte Carlo algorithm. On the other hand, the collapse cone convolution algorithm calculated the dose of the HT treatment plans with the dose grid of 3.12 mm and 1.27 ± 0.33 mm by the Hi‐Art and Precision, respectively. The plan parameters of these modalities were summarized in Table 1. The prescribed dose was dependent on the location and size of the PTV. The radiation dose of the PTV was in the range of 30 Gy ‐ 50 Gy (46.1 ± 6.6 Gy and 50.0 Gy, Mean ± SD and Median) in four to five fractions (Fxs). This dose was delivered on the various sizes of the PTV with volume in the range of 14.6 cc ‐ 225.7 cc (60.8 ± 53.9 cc and 37.0 cc). The prescribed dose was constrained to cover the target by at least 95% of the volume. The limited dose was also constrained on the liver, duodenum, esophagus, stomach, bowel, both kidneys, and Planning organ at Risk Volume (PRV) of the spinal cord as per the recommendation. The PRV of the spinal cord was 5 mm expanded from the spinal cord as well. The ring of 20 mm was expanded from the PTV. This virtual organ was created for the conformity of the prescribed dose. Other plan parameters depended on each planner such as the collimator angles, degrees of rotation, numbers of rotation, pitch, modulation factor, field width, either directional block or exit only of the leaf opening, etc. Although various plan parameters were used, but their focus was on the dosimetric parameters that met the guideline.

**TABLE 1:** Characteristics of patients and treatment plannings.

	Dx	PTV (cc)	Segment	Target dose (Gy)	Fxs	Modality	Arc (No.)	Arc (degree)	FWd (mm)	Pitch	act. MF
1	HCC	14.6	5	50	5	VMAT	2	200/200			
2	HCC	19.4	3	50	5	VMAT	2	360/360			
3	HCC	19.8	7	50	5	VMAT	2	360/360			
4	HCC	23.9	8	50	5	VMAT	2	360/360			
5	HCC	28.1	8	50	5	VMAT	2	360/360			
6	HCC	29.0	2,3	40	5	VMAT	2	180/180			
7	HCC	30.4	6	50	5	VMAT	2	360/360			
8	HCC	44.4	3,4	40	5	VMAT	2	260/260			
9	HCC	48.7	7,8	50	5	VMAT	2	270/270			
10	HCC	57.0	6,7	40	5	VMAT	3	45/170/35			
11	HCC	94.9	4	50	5	VMAT	2	360/360			
12	HCC	117.0	6,7	50	5	VMAT	2	300/90			
13	HCC	121.2	8	35	5	VMAT	2	360/360			
14	HCC	225.7	8	30	5	VMAT	2	360/360			
15	Liver metastasis	16.4	5	30	5	HT			25	0.215	1.399
16	HCC	22.1	2	50	5	HT			25	0.215	1.591
17	HCC	25.4	8	50	5	HT			25	0.125	1.568
18	HCC	29.1	8	50	5	HT			25	0.125	1.289
19	HCC	34.8	2,3	50	5	HT			50	0.287	1.654
20	HCC	37.0	6,7	50	5	HT			50	0.143	2.360
21	HCC	53.0	8	50	5	HT			50	0.200	1.500
22	HCC	64.1	8	50	5	HT			25	0.215	2.000
23	Liver metastasis	83.1	5	48	4	HT			25	0.287	1.800
24	HCC	95.4	3	40	5	HT			50	0.215	1.547
25	HCC	185.4	8	50	5	HT			25	0.287	1.500
Mean		60.8	–	46.1	5.0	–	2.1	–	34.1	0.210	1.655
SD		53.9	–	6.6	0.2	–	0.3	–	12.6	0.061	0.301
Median		37.0	–	50.0	5.0	–	2.0	–	25.0	0.215	1.568
Min		14.6	–	30.0	4.0	–	2.0	–	25.0	0.125	1.289
Max		225.7	–	50.0	5.0	–	3.0	–	50.0	0.287	2.360

**Abbreviations**: Dx, Diagnosis; PTV, Planning Target Volume; Fx, Fraction; FWd, Field Width with dynamic jaws; act. MF, actual Modulation Factor; HCC, Hepatocellular carcinoma; VMAT, Volumetric Modulated Arc Therapy; HT, Helical Tomotherapy; SD, Standard Deviation; Min, Minimum and Max, Maximum.

### 2.4 Dosimetric parameters

The SABR technique delivers a high radiation dose on the target. The limited dose of the OARs is then interpreted in the small volume of each organ. The dose on the duodenum, esophagus, stomach, bowel, and PRV spinal cord was defined by a volume of 0.05 cc (D_0.05cc_) whereas the kidneys were the mean dose (D_mean_). The liver is the main organ that requires consideration. The remaining liver (liver_rem_) was reconstructed by the subtraction between the whole liver and the Gross Target Volume (GTV). The liver_rem_ dose was indicated by D_mean_ and percent volume at 10 Gy (V_10Gy_). The surrounding dose is one of the main dosimetric parameters of interest. This parameter was indicated by the absolute volume at 30 Gy (V_30Gy_). Finally, the location of the PTV was indicated by the segment of the liver.

Accordingly, these are the dosimetric parameters that were guided by RTOG‐1112. However, some other parameters and indexes were evaluated. Although the name, definition, and ideal value of each index are summarized in Table 2, the details of each index are elucidated.

**TABLE 2:** Mathematical definition of plan quality metrics.

Dosimetric index	Definition	Ideal value	References
Homogeneity	$\mathrm{HI}=\;D_{\max}/D_{\mathrm{Rx}}$	1	RTOG^13^
Conformity	$CI_{\mathrm{Paddick}}=\;{\mathrm{TV}}_{\mathrm{PIV}}^{2}/\left(\mathrm{PTV}\times \mathrm{PIV}\right)$	1	Paddick^15^
	$CI_{\mathrm{ICRU}}=\mathrm{PIV}/\mathrm{PTV}$	1	ICRU83^16^
	$CI_{50\%}=\mathrm{PI}V_{50\%}/\mathrm{PTV}$	*Remark	RTOG 0915^17^
	$V_{50,100}=\;\mathrm{PI}V_{50\%}/\mathrm{PI}V_{\mathrm{Rx}}$	*Remark	Paddick and Lippitz^18^
Gradient distance	$\mathrm{GD}=\;\sqrt[{3}]{3\mathrm{PI}V_{50\%}/4\pi }-\sqrt[{3}]{3\mathrm{PIV}/4\pi }$	*Remark	Wagner et al.^19^
	$R_{50,100}=\;\left(\sqrt[{3}]{3\mathrm{PI}V_{50\%}/4\pi }\;\right)\;/\;\left(\sqrt[{3}]{3\mathrm{PIV}/4\pi }\right)$	*Remark	Wagner et al.^19^
Integral dose	$\mathrm{ID}=D_{\mathrm{mean}}\times \;\mathrm{Volume}$	As low as possible	Snyder et al.^22^

^*^Remark = Should be as low as possible or depend on the individual treatment center agreement.

**Abbreviations**: HI = Homogeneity index; D_max_ = Maximum dose; D_Rx_ = Target dose; CI_ICRU_ = Conformity index of ICRU; CI_Paddick_ = Conformity index of Paddick; PIV, Prescription isodose volume; PTV, Planning target volume; TV, Treated volume; CI_50%_, Conformity index at 50% isodose level of the prescribed dose; PIV_50%_, 50% isodose level of the prescribed dose; V_50,100_, Isodose volume ratio between the dose level of 50% and prescribed dose; GD, Gradient distance; R_50,100_, Effective distance ratio between the dose level of 50% and the prescribed dose; ID, Integral dose and D_mean_, mean dose.

**Homogeneity Index** ^13^ **(HI)** is the most considered index of treatment planning. This index shows the dose homogeneity in the target and is calculated by D_max_/D_Rx_. The D_max_ is the maximum dose and D_Rx_ is the prescribed dose. There are various formulas of the HI^14^ that are used in radiation therapy. This study provided the dose of various volumes that might apply in these indexes (see Supplementary Table 1).

**The conformity Index (CI)** interprets the prescribed dose on the target. Two formulas of this index are from Paddick^15^ and the International Committee on Radiation Units and measurements^16^ (ICRU). The CI formalism of ICRU (CI_ICRU_) accounts only for the volume between the target and prescribed dose but not for the intersection between those two volumes as mentioned by the CI formalism of Paddick (CI_Paddick_). The CI_ICRU_ was calculated by PIV/PTV where PIV is the prescribed isodose volume. The CI_Paddick_ was calculated by ${\mathrm{TV}}_{\mathrm{PIV}}^{2}/\left(\mathrm{PTV}\times \mathrm{PIV}\right)$ where the TV_PIV_ is the PIV of the target.

**The conformity index at 50% of PIV** ^17^ **(CI_50%_)** shows the area of the dose at 50% of the target dose. The formalism of this index was calculated as it was the CI_ICRU_, but the PIV is the PIV_50%_. The value was calculated by $\mathrm{PI}V_{50\%}/\mathrm{PTV}$. **V_50,100_** ^18^ is the ratio between the volume of the prescribed dose and the dose at 50% of the prescription. This ratio was calculated for the isodose volume proportion.

**Gradient Distance (GD)** identifies the distance of the dose gradient. This value was modified from the formalism of the conformity gradient index^19^ (CGI) as was evaluated in the intracranial stereotactic radiosurgery.^20^, ^21^ The effective radius (R_eff_) was calculated by the volume of the two isodose levels, prescribed isodose level (PIV) and 50% of prescribed isodose level (PIV_50%_), and subtraction of each other. This index is then calculated by $\sqrt[{3}]{3\mathrm{PI}V_{50\%}/4\pi }-\sqrt[{3}]{3\mathrm{PIV}/4\pi }$. **R_50,100_** ^19^ is the ratio between the effective radius of the volume between the prescribed dose and the dose at 50% of the prescription. This ratio was calculated for the proportion of the effective radius by $\left(\sqrt[{3}]{3\mathrm{PI}V_{50\%}/4\pi }\;\right)\;/\;\left(\sqrt[{3}]{3\mathrm{PIV}/4\pi }\right)$.

**Integral Dose** ^22^ **(ID)** shows the low dose volume that distributes on the patient's body. This value was calculated by D_mean_ × Volume where the Volume is the body captured in the entire CT scan. This parameter has a unit of Gy⋅L.

### 2.5 Statistical analysis

This study analyzed the plan quality of SABR in the liver tumor. However, the patients were separated into two groups according to the treatment modalities. The performance between treatment modalities was then compared and analyzed by utilizing the dosimetric parameters and indexes. The SPSS version 25 (IBM Co., New York, USA) analyzed the normal distribution of the data by utilizing the Shapiro‐Wilk test. The independent *t*‐test analyzed the data of the normal distribution whereas the Mann‐Whitney *U* test analyzed the data of the non‐normal distribution. The statistical analysis tested all data with a threshold of 0.05 *P*‐value.

## 3 RESULT

### 3.1 Dosimetric treatment planning analysis

The plan quality of the SABR for liver tumors is revealed by the dosimetric parameters. The result reveals that the prescribed dose covered the volume of PTV by 97.3 ± 3.9%. This coverage has the value of HI, CI_Paddick_, CI_ICRU,_ and CI_50_ at 1.1 ± 0.0, 0.8 ± 0.1, 1.2 ± 0.1, and 5.3 ± 1.1, respectively. The GD is 1.5 ± 0.3 cm indicates the distance of the dose level between the prescribed dose and 50% of the prescribed dose. Two parameters demonstrate the dose on the liver_rem_. This organ receives the mean radiation dose at 10.4 ± 4.0 Gy whereas the 38.2 ± 16.8 cc of its volume receives the dose at 10 Gy. The dose of 0.05 cc can represent the D_max_ of the organ. These plans present the mean of D_max_ on the duodenum, esophagus, bowel, stomach, and PRV spinal cord, which are 12.0 ± 11.1 , 11.6 ± 6.7 , 11.4 ± 9.5 , 14.6 ± 8.4, and 9.9 ± 5.7 Gy, respectively. Additionally, the mean of D_mean_ on kidneys is 2.8 ± 2.7 Gy. The spread out of the dose can be observed by the volume of the surrounding dose at 30 Gy and ID. The plans show the mean of surrounding dose volume and ID which are 175.6 ± 130.0 cc and 38.5 ± 16.1 Gy⋅L, respectively.

### 3.2 Dosimetric treatment planning comparison

Treatment planning was separately performed by two treatment modalities. The results of the separate modalities are illustrated in Table 3. Although no significant difference in the target size between these two techniques were observed, the group of HT has a slightly smaller volume of the PTV than the group of VMAT, in term of the mean. The prescribed dose between these techniques was observed as well. The statistical analysis shows no significant difference in the prescribed dose between the VMAT and HT. In contrast to the PTV sizes, the radiation dose delivered to the target through the HT is slightly higher than the VMAT. Other plan parameters have no significant difference between these two modalities as indicated by the indexes of the HI, CI_ICRU,_ and GD and the doses on the PTV, liver_rem_, duodenum, esophagus, kidneys, stomach, PRV spinal cord, surrounding dose volume, and ID. Although there is no significant difference between the VMAT and HT, the HT technique shows a slightly higher dose on the esophagus and kidneys than the VMAT whereas the value of CI_ICRU_, V_50,100_, GD, and R_50,100_, and the dose on liver_rem_, duodenum, and PRV spinal cord are lower. The surrounding dose and ID have the opposite direction of the values. The HT provides the higher value of the surrounding dose whereas the value of ID is lower than the VMAT. The significant difference between the two techniques was observed on the CI_Paddick_, CI_50,_ and the dose on the bowel. The treatment plan of the VMAT shows the value of CI_Paddick_ less than the HT (*P* = 0.002), whereas the value of CI_50_ is higher (*P* = 0.032). Finally, the treatment plans of the VMAT deliver the dose to the bowel more than the treatment plans of the HT.

**TABLE 3:** The dosimetric parameters, indexes, dose constraints, and dose on OARs of all treatment and separated treatment modalities.

	Parameter, (unit)	RTOG constraints	All modalities	HT	VMAT	*P*
PTV	(cc)		60.8 ± 53.9	58.7 ± 49.2	62.4 ± 59.1	
	V_100%_, (%)	V_100%_ ≥ 95%	97.3 ± 3.9	97.4 ± 2.3	97.3 ± 4.9	
Prescribed dose	(Gy)	–	46.1 ± 6.6	47.1 ± 6.4	45.4 ± 6.9	
HI		–	1.1 ± 0.0	1.1 ± 0.0	1.1 ± 0.1	
CI_Paddick_		–	0.8 ± 0.1	0.9 ± 0.1	0.7 ± 0.2	= 0.002
CI_ICRU_		–	1.2 ± 0.1	1.1 ± 0.1	1.2 ± 0.2	
CI_50_		–	5.3 ± 1.1	4.8 ± 0.6	5.7 ± 1.2	= 0.032
V_50,100_		–	4.4 ± 0.6	4.2 ± 0.4	4.6 ± 0.7	
GD	(cm)	–	1.5 ± 0.3	1.4 ± 0.3	1.6 ± 0.3	
R_50,100_		–	1.6 ± 0.1	1.6 ± 0.1	1.7 ± 0.1	
Liver_rem_	(cc)	–	1042.8 ± 257.9	953.4 ± 186.4	1113.1 ± 289.8	
	D_mean_, (Gy)	Depend on the prescribed dose	10.4 ± 4.0	10.0 ± 4.2	10.8 ± 4.0	
	V_10Gy_, (%)		38.2 ± 16.8	35.2 ± 16.8	40.5 ± 17.1	
Duodenum	D_0.05cc_, (Gy)	≤ 30.0	12.0 ± 11.1	11.7 ± 14.4	12.1 ± 10.4	
Esophagus	D_0.05cc_, (Gy)	≤ 32.0	11.6 ± 6.7	12.1 ± 8.7	11.2 ± 5.1	
Kidneys	D_mean_, (Gy)	≤ 10.0	2.8 ± 2.7	3.0 ± 2.9	2.4 ± 2.8	
Bowel	D_0.05cc_, (Gy)	≤ 30.0	11.4 ± 9.5	3.4 ± 2.9	17.4 ± 7.9	= 0.034
Stomach	D_0.05cc_, (Gy)	≤ 30.0	14.6 ± 8.4	14.6 ± 10.2	14.6 ± 7.5	
PRV spinal cord	D_0.05cc_, (Gy)	≤ 25.0	9.9 ± 5.7	9.0 ± 4.7	10.6 ± 6.4	
Surrounding dose	V_30Gy_, (cc)	–	175.6 ± 130.0	187.2 ± 173.1	166.5 ± 89.3	
Integral dose	(Gy⋅L)	–	38.5 ± 16.1	36.3 ± 15.8	40.1 ± 16.7	

**Abbreviations**: HT, Helical tomotherapy; VMAT, Volumetric modulated arc therapy; PTV, Planning target volume; HI, Homogeneity index; CI_Paddick_ , Conformity index of Paddick; CI_ICRU_ , Conformity index of ICRU; CI_50_, Conformity index at 50% prescribed dose; V_50,100_, Volume ratio between 50% and 100% isodose level; GD, Gradient distance; R_50,100_, Distance ratio between effective distance of 50% and 100% isodose level; Liver_rem_, Remaining liver and PRV, Planning organ at risk volume.

## 4 DISCUSSION

### 4.1 Dosimetric treatment planning analysis

SABR is one of the advanced radiation techniques that deliver a highly accurate radiation dose to patients. The RTOG‐1112 provides the guideline of the dose on target and OARs for the SABR in the liver tumor. Our treatment plans reveal the dose constraints that have passed the criteria of the guideline such as the target coverage and dose on OARs. Although there are no recommended parameters of the plan quality, the parameters of the stereotactic radiosurgery then were utilized in the plan quality assessment.

**FIGURE 1:**
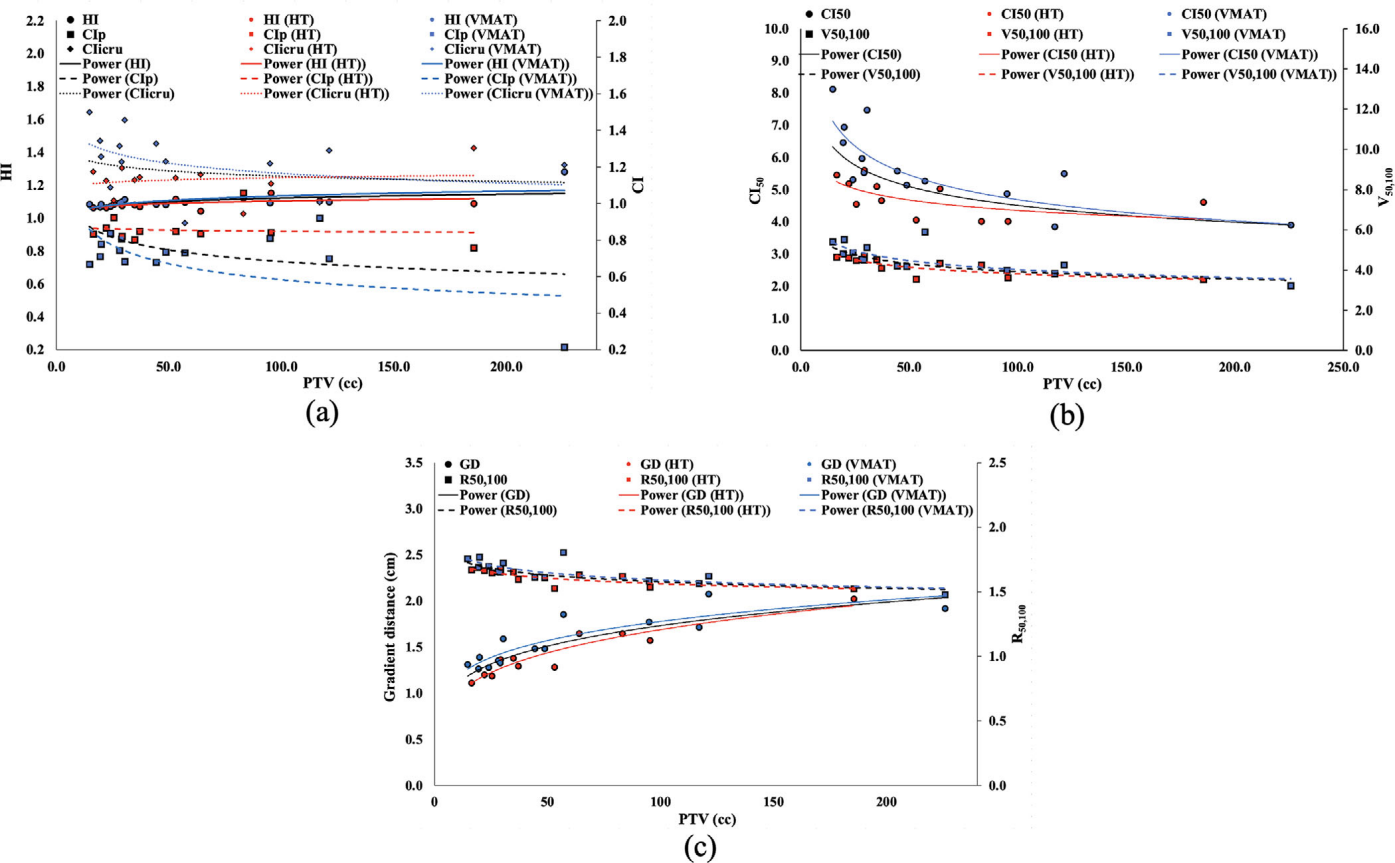
Plots and trend lines between the size of the PTV and various dosimetric parameters: (a) The size of PTV versus HI, CI_ICRU,_ and CI_paddick_ are presented in the dots and lines, the diamond squares and roe lines, and the boxes and dash lines, respectively. (b) The size of PTV versus CI_50_ and V_50,100_ is presented in the dots and lines, and boxes and dash lines, respectively. (c) The size of PTV versus GD and R_50,100_ is presented in the dots and lines, and boxes and dash line, respectively.

The HI is one of the dosimetric parameters that are under consideration. The mean value of the HI made the treatment plan more impressive with less deviation in conformity with the work of various publications.^5^, ^6^, ^7^ These values then were plotted against the size of PTV as demonstrated in Figure 1a. The consistent value along the size of the PTV revealed parallel results with the work of Paik et al.^5^ The CI is the index that shows the target coverage of the prescribed dose. The CI_ICRU_ is normally utilized for the evaluation of conformity in the SABR^5^, ^7^ of the liver tumor. This value, 1.2 ± 0.1, shows the conformity is slightly superior with an increase in the size of PTV as observed in Figure 1a. The CI_Paddick_ is one of the effective formalisms that consider the intersected volume between the prescribed dose and the target. The value, 0.8 ± 0.1, decreases with the size of the PTV as given in Figure 1a. In contrast to the CI_ICRU_, this conformity is inferior with the increase in the size of the PTV. According to the value of CI_ICRU_, the value is slightly consistent with an increase in the size of PTV. The PIV and PTV are perhaps the parallel increasing volume; moreover, these two parameters are the denominator of the CI_Paddick_. The TV_PIV_ of the CI_Paddick_ formalism was restricted by the target constraints, thus this term can assume to be the consistent value. The increment of the denominator then directly impacted the value of CI_Paddick_. This might show that the formalism of CI_Paddick_ is more sensitive to the evaluation of conformity than the CI_ICRU_.

The CI_50_ evaluated the volume of the radiation dose on the adjacent normal tissue. The value decreases along with an increase in the size of the PTV as illustrated in Figure 1b. This reveals the dose spread‐out ratio in the small targets is larger than in the large targets. Although the spread of the dose was observed through the value of CI_50_, the value of V_50,100_ (in Figure 1b) shows the proportion of the volume between these two doses that are not slightly different along various sizes of the PTV. Accordingly, the dose spreads out then appear in the small target rather than the large one. The distance of the gradient is another parameter that can observe the dose spread out. The value of the GD is larger with the increase in the size of the PTV as presented in Figure 1c. The R_50,100_ demonstrates a slightly consistent value along with increasing sizes of the PTV as shown in Figure 1c. This reveals that the value of the GD is influenced by PTV size rather than the PIV.

**FIGURE 2:**
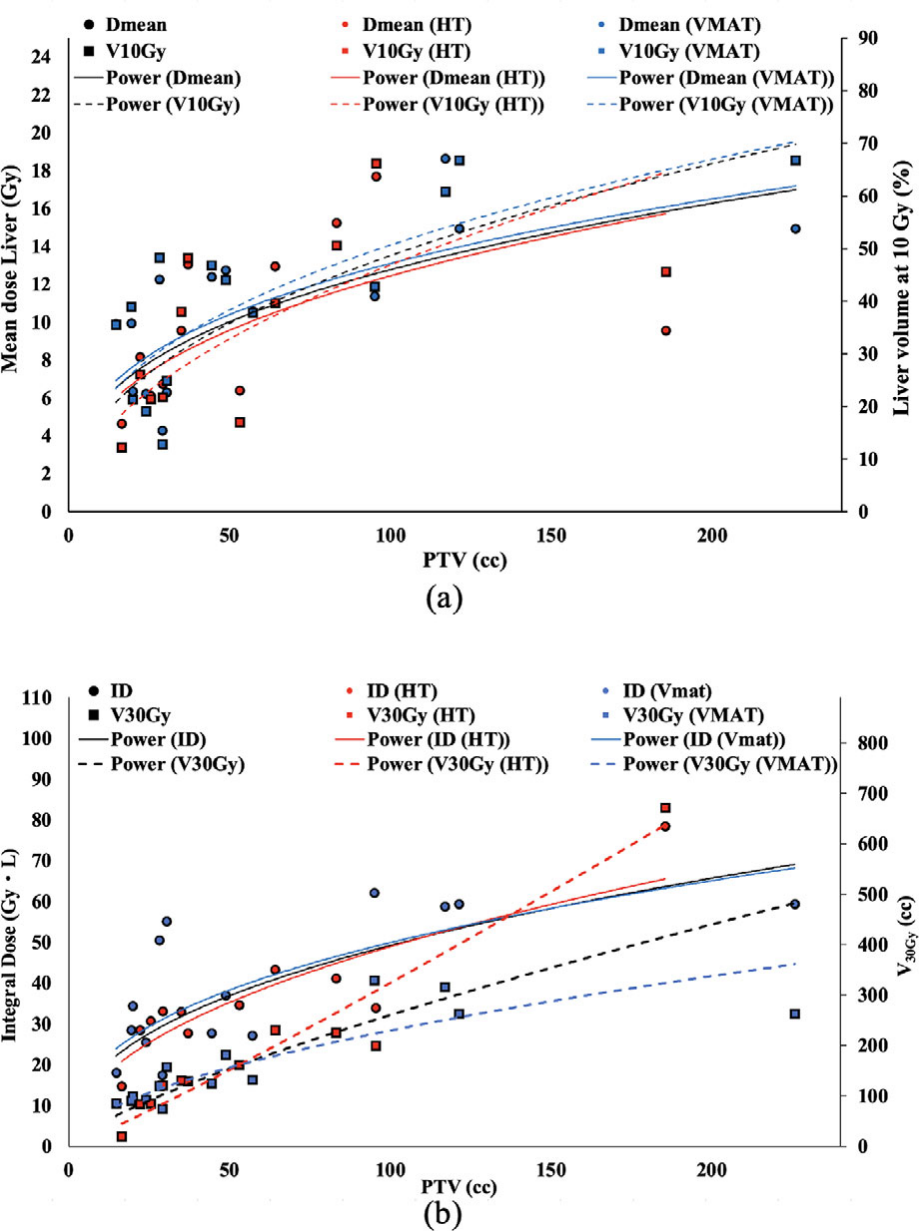
Plots and trend lines between the size of the PTV and various dosimetric parameters of the OARs: (a) The size of PTV versus D_mean_ and V_10Gy_ of Liver_rem_ is presented in the dots and lines, and the boxes and dash line, respectively. (b) The size of PTV versus ID and V_30Gy_ is presented in the dots and lines, and the boxes and dash lines, respectively.

Due to the lesion in the liver, the liver_rem_ is the most concerning organ of radiation delivery. The treatment focuses on liver function because of the low radiation dose tolerance. The guideline recommended criteria is that the liver_rem_ should have a volume larger than 700 cc, excluding the GTV. The liver_rem_ was analyzed by the plot between the dose and the volume of the liver and the size of PTV as demonstrated in Figure 2a. This figure shows that the liver_rem_ receives the increasing radiation dose as well as the volume of the organ, along with the size of PTV. The OARs, such as the duodenum, esophagus, kidneys, bowel, stomach, and PRV spinal cord, are surrounding the liver. The dose on each normal organ thus depends on the location of the PTV in the liver. This tumor location was classified by the segment of the liver.^23^ In case the lesion is in two segments, the target location is expressed in terms of segment x.5 such as 2.5 (The tumor location between segment 2 and segment 3). The analysis concentrates the dose on each OAR of each tumor location as illustrated in Supplementary 2. The high dose delivers to the duodenum, bowel, and kidneys is when the tumor is located in the middle part of the liver segment, from segment 4 to segment 7, whereas the esophagus receives the low dose in this area. The stomach and PRV spinal cord obtain the high dose where the tumor is located slightly to the end of the liver segment, segment 6 to segment 8. However, the dose of each OAR is dependent on the treatment techniques. The results of this study were reported by employing the radiation technique of Intensity Modulated Arc Therapy (IMAT). The dose on OARs required more investigation when other treatment techniques were employed such as the 3D‐CRT, static gantry IMRT, CK, etc. The ID is a famous topic of discussion when the technique of IMAT was utilized. The ID and V_30Gy_ were plotted against the size of the PTV as illustrated in Figure 2b. This is straightforward that these two parameters are increasing along with the increasing volume of PTV.

### 4.2 Dosimetric treatment planning comparison

The guideline provides various techniques and treatment modalities for the SABR in liver tumor treatment. Our center utilized two treatment techniques on two treatment modalities in this manner. Dose delivery through the VMAT technique utilized the C‐arm‐based Linac whereas the ring‐based Linac of the TomoTherapy delivered the dose of the HT technique. According to the small groups of the sample size, the statistics analyzed the significant difference between these groups. To prevent statistical bias, the target size, dose prescription, and liver_rem_ were analyzed and confirmed. The analysis of the target size, dose prescription, and liver_rem_ demonstrated no significant difference between these two groups of treatment techniques. On the HI, the mean value reveals less deviation in conformity to various publications.^3^, ^5^, ^6^, ^7^, ^8^ The consistent value with the increasing size of PTV is observed on both techniques, presented in Figure 1a, and in line with the result of Thaper et al.^3^ and Paik et al.^5^ The less variation of this parameter is sporadically observed due to control by the planners. In the CI_ICRU_, the mean value of VMAT shows lower performance than the HT and is in line with the result of Choi et al.^7^ However, the VMAT provides a higher value only in the small volume PTV than the HT whereas it contrasts with the large target volume as presented in Figure 1a. On the other hand, the HT provides the consistent value of CI_Paddick_ along the increasing size of the PTV when the intersection volume between PIV and PTV is considered as observed in Figure 1a. The VMAT displays a large variety of the value when the target size is increasing which contrasts the result of Oymak et al.^6^ They found less variation on both C‐arm‐based and ring‐based Linac but the HT revealed significantly higher performance than others. The dose calculation grid is one of the treatment plan parameters that might have an impact on the CI.^24^ The grid of the VMAT plans was employed by 3.00 mm spacing whereas the HT was two grid sizes spacing: 1) 3.12 mm spacing on the Hi‐Art and 2) 1.27 ± 0.33 mm spacing on the Precision. The grid size of the Tomotherapy treatment planning was selected by Fine, Medium, and Coase, and calculated by FieldofView/NumbersofPixel. The difference between the Hi‐Art and the Precision is the number of pixels. The calculation of Fine in the Precision utilized the image matrix of 512 × 512 pixels, whereas the Hi‐Art employed the matrix of 256 × 256 pixels. Due to the comparable grid size, this study then continuously investigated the comparison of the CI_Paddick_ between the VMAT and HT by eliminating the plan of the Precision. The value of CI_Paddick_ was 0.8 ± 0.1 for the plan of the Hi‐Art. The statistics analyzed the significant difference between these two groups of the treatment plan and found significantly lower performance of the VMAT (0.7 ± 0.2, *P* = 0.008) than the HT in terms of CI_Paddick_. For CI_50_, the parallel result of the CI_ICRU_ was observed in this value but it is different on the large volume of the target. Both techniques present a comparable performance of the dose gradient in the large target confirming the results from the work of Thaper et al.^3^ The low value of the CI_50_ represents the high dose gradient between the PTV and the PIV_50%_. This roughly reveals that the VMAT might have a lower resolution of the intensity modulation when compared with the HT in terms of the co‐planar technique, particularly in the small PTV, as demonstrated in Figure 1b.

The dose spread out is an interesting point of discussion as observed in Figure 2b. The value of the ID is well parallel to each other technique but not on the V_30Gy_. The formalism of the ID was calculated by utilizing the D_mean_ that perhaps less the sensitivity than the specific level of the radiation dose. The dose delivered to the target through the HT is slightly higher than the VMAT. These might be the reason that magnified the sensitivity of the V_30Gy_ on the HT technique as illustrated in Figure 2b. Although the difference was observed, the statistics presented no significant difference in both ID and V_30Gy_ between these two techniques. This might cause that no significant difference was shown on all OARs except the bowel. On the bowel, the VMAT provides a radiation dose on this organ higher than the HT. This result is in contrast to the work of Oymak et al.^6^ As previously mentioned, the dose on the surrounding organs could depend on the target location. Some segment of the liver, particularly segment 4–6, is within reach of the bowel. The samples have the target location at these segments which are six cases in the technique of VMAT. This could provide the deviation of the statistical dosimetric comparison between these two techniques. The dosimetric comparison of the SABR in a specific location of the liver tumor among the treatment modalities may require more investigation.

## 5 CONCLUSION

The SABR is one of the treatment techniques that is available for liver tumors. Although providing quality guidelines for treatment planning, the dosimetric parameters and indexes should be utilized for the plan evaluation. The various modalities were employed for treatment; however, both can achieve the treatment criteria with slightly low performance on the C‐arm‐based Linac. Finally, the dose of OARs might be highly dependent on the tumor location. Furthermore, this study might provide the information to set up the plan quality check for the SABR in the liver tumor.

## AUTHOR CONTRIBUTIONS

Anirut Watcharawipha: Conceptualization, Investigation, Formal analysis, Writing—original draft, Writing—review and editing., Somvilai Chakrabandhu: Conceptualization, Formal analysis, Writing—review and editing, Supervision., Anupong Kongsa: Formal analysis, Data curation., Damrongsak Tippanya: Formal analysis, Data curation., Imjai Chitapanarux: Formal analysis, Writing—review and editing.

## CONFLICT OF INTEREST STATEMENT

This research has no conflict of interest.

## Supporting information


**Supplementary 1**. PTV, prescribed dose and dose level of various PTV volumes.

**Supplementary 2**. Plots and trend lines of polynomial order 2 fitting curves between the PTV location in the liver and the dose on OARs: a) The liver segments versus the dose on the duodenum, esophagus and kidneys. b) The liver segment versus the dose on the bowel, stomach and PRV spinal cord. The large symbols represent the mean dose of each organ whereas the small symbols are the dose on the organs of each case.
