# Supplementary material for: Plan quality analysis of stereotactic ablative body radiotherapy treatment planning in liver tumor
Source: J Appl Clin Med Phys. 2023 Mar 1;24(7):e13948. doi: 10.1002/acm2.13948 (PMC10338832; doi:10.1002/acm2.13948)
Supplement: Supplementary file 1 — Supplementary 1. PTV, prescribed dose and dose level of various PTV volumes. [file ACM2-24-e13948-s002.docx]

| **Supplementary 1** PTV, prescribed dose and dose level of various PTV volumes | | | | | | | | | | | | | |
| --- | --- | --- | --- | --- | --- | --- | --- | --- | --- | --- | --- | --- | --- |
| No. | PTV  (cc) | Prescribed dose | D_min_ | D_2cc_ | D_1%_ | D_2%_ | D_5%_ | D_50%_ | D_90%_ | D_95%_ | D_99%_ | D_max_ |  |
|  |  | (Gy) | | | | | | | | | | |  |
| ***Volumetric Modulated Arc Radiotherapy*** | | | | | | | | | | | | | |
| 1 | 14.6 | 50.0 | 50.1 | 52.3 | 53.0 | 52.8 | 52.6 | 51.8 | 51.2 | 51.0 | 50.7 | 54.2 |  |
| 2 | 19.4 | 50.0 | 45.8 | 52.3 | 52.8 | 52.7 | 52.5 | 51.8 | 51.1 | 50.8 | 48.9 | 53.4 |  |
| 3 | 19.8 | 50.0 | 47.9 | 52.9 | 53.4 | 53.2 | 53.0 | 52.2 | 51.1 | 50.7 | 49.9 | 54.2 |  |
| 4 | 23.9 | 50.0 | 46.3 | 52.4 | 52.9 | 52.8 | 52.6 | 51.6 | 50.6 | 50.1 | 48.9 | 53.7 |  |
| 5 | 28.1 | 50.0 | 43.8 | 52.8 | 53.5 | 53.3 | 52.9 | 52.0 | 51.2 | 50.9 | 49.9 | 54.6 |  |
| 6 | 29.0 | 40.0 | 38.2 | 42.4 | 42.9 | 42.7 | 42.5 | 41.7 | 41.2 | 41.0 | 40.4 | 44.0 |  |
| 7 | 30.4 | 50.0 | 48.6 | 53.9 | 54.6 | 54.3 | 54.0 | 52.8 | 51.9 | 51.6 | 50.9 | 55.7 |  |
| 8 | 44.4 | 40.0 | 37.5 | 42.3 | 42.6 | 42.5 | 42.3 | 41.6 | 40.9 | 40.6 | 39.8 | 43.3 |  |
| 9 | 48.7 | 50.0 | 45.2 | 52.5 | 52.8 | 52.6 | 52.6 | 51.8 | 51.2 | 50.9 | 50.0 | 54.1 |  |
| 10 | 57.0 | 40.0 | 19.6 | 42.2 | 42.5 | 42.3 | 42.1 | 41.0 | 38.7 | 34.2 | 26.3 | 43.9 |  |
| 11 | 94.9 | 50.0 | 47.1 | 53.3 | 53.5 | 53.3 | 53.0 | 51.9 | 51.1 | 50.8 | 50.2 | 54.6 |  |
| 12 | 117.0 | 50.0 | 43.6 | 54.2 | 54.3 | 54.1 | 53.8 | 52.4 | 50.9 | 50.3 | 47.5 | 55.3 |  |
| 13 | 121.2 | 35.0 | 33.1 | 37.4 | 37.5 | 37.3 | 37.1 | 36.5 | 35.9 | 35.6 | 35.1 | 38.5 |  |
| 14 | 225.7 | 30.0 | 27.1 | 37.4 | 37.3 | 37.2 | 37.0 | 35.9 | 32.3 | 31.2 | 29.3 | 38.4 |  |
| ***Helical Tomotherapy*** | | | | | | | | | | | | | |
| 15 | 16.4 | 30.0 | 28.9 | 31.4 | 31.8 | 31.7 | 31.5 | 30.9 | 30.5 | 30.4 | 30.0 | 32.4 |  |
| 16 | 22.1 | 50.0 | 48.9 | 52.5 | 52.9 | 52.8 | 52.6 | 51.9 | 50.8 | 50.6 | 50.0 | 53.3 |  |
| 17 | 25.4 | 50.0 | 46.1 | 53.5 | 53.9 | 53.8 | 53.6 | 52.3 | 50.9 | 50.4 | 49.3 | 54.2 |  |
| 18 | 29.1 | 50.0 | 49.0 | 52.6 | 53.3 | 53.1 | 52.7 | 51.7 | 50.6 | 50.5 | 50.2 | 53.8 |  |
| 19 | 34.8 | 50.0 | 48.8 | 53.8 | 54.0 | 53.9 | 53.9 | 52.8 | 50.2 | 50.0 | 49.8 | 54.1 |  |
| 20 | 37.0 | 50.0 | 48.6 | 52.4 | 52.9 | 52.6 | 52.4 | 51.2 | 50.7 | 50.5 | 49.7 | 53.5 |  |
| 21 | 53.0 | 50.0 | 45.1 | 54.0 | 54.4 | 54.2 | 53.9 | 52.6 | 51.6 | 51.1 | 49.4 | 55.8 |  |
| 22 | 64.1 | 50.0 | 45.6 | 54.0 | 54.6 | 54.2 | 53.9 | 52.1 | 51.0 | 50.7 | 49.7 | 56.7 |  |
| 23 | 83.1 | 48.0 | 42.8 | 53.3 | 53.5 | 53.4 | 53.1 | 50.5 | 48.1 | 47.5 | 45.8 | 53.9 |  |
| 24 | 95.4 | 40.0 | 34.9 | 45.2 | 45.4 | 45.2 | 44.8 | 43.7 | 40.9 | 40.5 | 38.5 | 46.2 |  |
| 25 | 185.4 | 50.0 | 49.8 | 53.5 | 53.5 | 53.4 | 53.3 | 52.0 | 50.6 | 50.5 | 50.3 | 54.5 |  |
| Abbreviation: PTV = Planning target volume, D_min_ = Minimum dose, D_2cc_ = Dose at 2 cc of PTV, D_X%_ = Dose at X% volume of PTV and D_max_ = Maximum dose. | | | | | | | | | | | | |  |
