# Supplementary material for: Plan quality analysis of stereotactic ablative body radiotherapy treatment planning in liver tumor
Source: J Appl Clin Med Phys. 2023 Mar 1;24(7):e13948. doi: 10.1002/acm2.13948 (PMC10338832; doi:10.1002/acm2.13948)
Supplement: Supplementary file 2 — Supplementary 2. Plots and trend lines of polynomial order 2 fitting curves between the PTV location in the liver and the dose on OARs: a) The liver segments versus the dose on the duodenum, esophagus and kidneys. b) The liver segment versus the dose on the bowel, stomach and PRV spinal cord. The large symbols represent the mean dose of each organ whereas the small symbols are the dose on the organs of each case. [file ACM2-24-e13948-s001.docx]

| **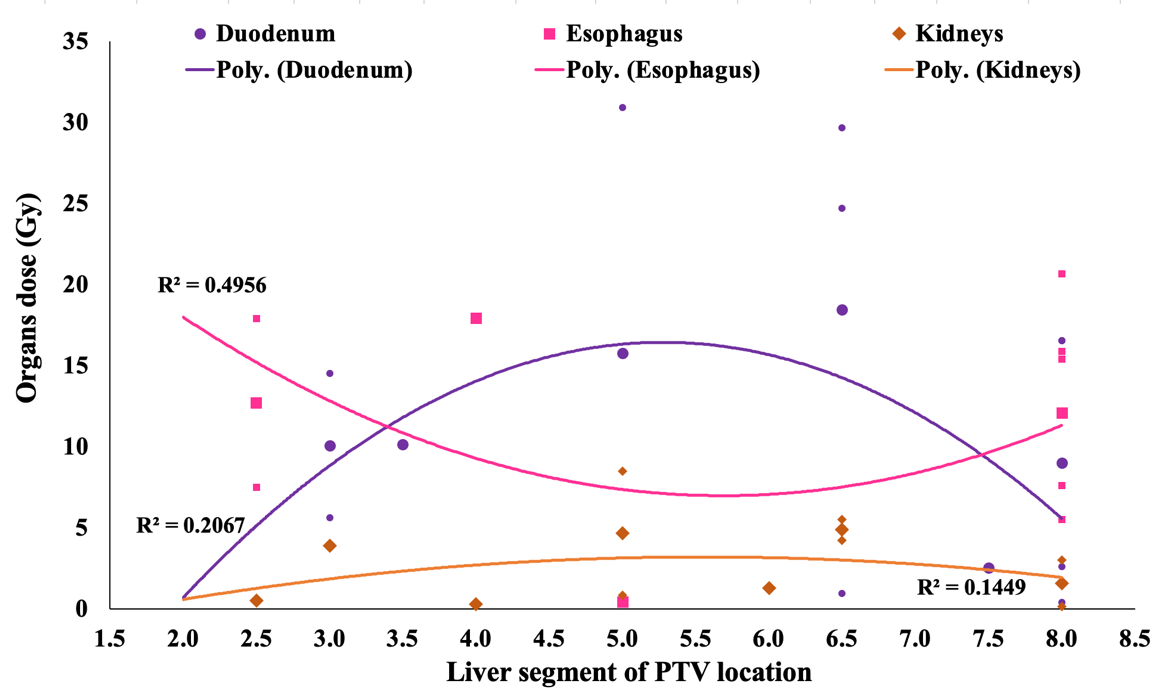**  (a) |
| --- |
| **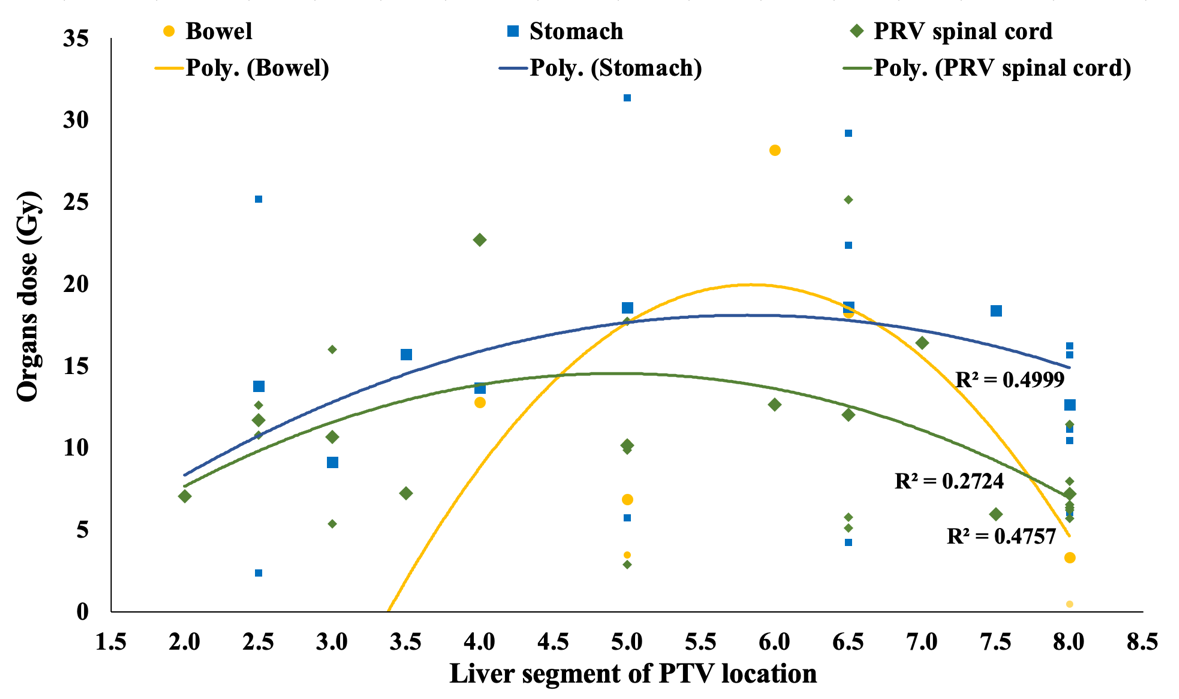**  (b) |
| **Supplementary 2.** Plots and trend lines of polynomial order 2 fitting curves between the PTV location in the liver and the dose on OARs: a) The liver segments versus the dose on the duodenum, esophagus and kidneys. b) The liver segment versus the dose on the bowel, stomach and PRV spinal cord. The large symbols represent the mean dose of each organ whereas the small symbols are the dose on the organs of each case. |
